# Study on the mechanism of urban morphology on river cooling effect in severe cold regions

**DOI:** 10.3389/fpubh.2023.1170627

**Published:** 2023-04-18

**Authors:** Fei Guo, Sheng Xu, Jun Zhao, Hongchi Zhang, Lijuan Liu, Zhen Zhang, Xinyuan Yin

**Affiliations:** ^1^School of Architecture and Fine Art, Dalian University of Technology, Dalian, Liaoning, China; ^2^Second Affiliated Hospital of Dalian Medical University, Dalian, Liaoning, China

**Keywords:** urban form factor, LST, river cooling effect, cold areas, spatial regression models

## Abstract

In the context of global warming, urban climate problems such as heat waves, urban heat islands and air pollution are becoming increasingly prominent, and the cooling effect of rivers is an effective way to mitigate urban hot climate. This study investigates the surrounding urban area of the Hun River in Shenyang, a severe cold region of China, by calculating satellite inversion surface temperature and urban morphology data, and explores the cooling effect of rivers using linear regression models and spatial regression models. The results show that (1) water bodies have a cooling effect on the surrounding environment, with the farthest cooling distance being 4,000 m, but the optimal cooling distance being 2,500 m. (2) In the results of the spatial regression model analysis, the *R*^2^ value stays above 0.7 in the range of 0–4,000 m, indicating that urban morphological factors are closely related to LST (land surface temperature). The negative correlation is most pronounced for NVDI (normalized vegetation index), with a peak of −14.8075 calculated by the regression model, and the positive correlation is most pronounced for BD (building density), with a peak of 8.5526. (3) The urban thermal environment can be improved and the heat island effect mitigated through measures such as increasing urban vegetation cover and reducing building density, and these findings can provide data references and case studies to support urban planning and development departments.

## 1. Introduction

In the context of global warming, the urban living environment is gradually deteriorating and the issue of urban heat islands and heat waves of high frequency, intensity and duration has become a widespread concern (1). High temperatures can cause cardiovascular, cerebrovascular and respiratory diseases; lead to business shutdowns, school closures and a reduction in outdoor recreational activities (2). High temperature weather also increases the use of air conditioning and water stress among urban residents, which also has a significant negative impact on energy conservation and emission reduction (3, 4). The older adult/adults is more vulnerable to high temperature and will be seriously affected by high temperature in the future. Therefore, it is very necessary to carry out health related assessment and urban planning to ameliorate heat risk in China, which is facing deep aging.

Several studies have shown that the human body adapts differently to the perception of the surrounding thermal environment in different regions (5–8). For example, people in colder regions are less able to adapt to heat, and the risk of increased mortality in hot summer conditions is much higher than in hotter regions (9, 10). Davis et al. analyzed more than 25 years of mortality data and found that most northern cities in the United States experienced excess summer mortality, in contrast to southern cities that experienced little or no increase in mortality during high temperatures, regardless of the severity of the extreme heat event (11). In the context of global warming, the occurrence of high temperatures in cold regions is increasing significantly in frequency. This provides new challenges for urban construction and planning.

The urban thermal environment is strongly influenced by building form, with different types of land use, building height, density and form all affecting the urban thermal environment to varying degrees (12). For example, B. Chun et al. found a positive correlation between LST and building roof area, and a negative correlation between NDVI, SVF and water body area and LST (13); Jun Yang finds that high-density high-rise buildings increase surface temperatures (14); Guanghua Guo et al. found that building density had a greater effect on LST than building height (15); Chun Yin et al. found that both land use type and urban form have a role in heat island mitigation (16). In addition, the urban form influences to some extent the ventilation efficiency of the city, which also contributes to the mitigation of urban heat islands (17, 18).

Water bodies are good regulators of the thermal environment and contribute significantly to high temperatures and urban heat islands (19). Rivers act as corridors to promote urban ventilation and the lower temperatures at the river surface have a cooling effect on the surrounding urban heat island (20). The intensity of the cooling effect is related to the size, area covered, form and distance of the river. For example, Cai Zhi et al. compared the spatial relationship between the layout of water bodies and the thermal effect of water bodies in 34 medium and mega cities in China, and concluded that for the majority of urban water bodies have a cooling effect and the average cooling distance is between 431 and 1,350 m (21); Saburo Murakaw et al. found that the Ota River in Hiroshima City cools in the direction of the vertical river up to a distance of about 100 m (22). Hathway et al. found that rivers can reduce surrounding temperatures by 1–1.5°C, with greater cooling achieved when connected to green spaces (23); Xu Xiyan and others found that the time when the water body has the best cooling effect on the surrounding area is around 14:00 in the afternoon (24). These studies all demonstrate the positive effect of rivers on urban heat island mitigation.

Existing studies on the thermal environment are more often based on localized investigations in different climatic zones by means of actual measurements (25–27), or a study on the thermal environment of regional landscapes near rivers (28–30). However, the impact of river cooling effects on the thermal environment has been less investigated at the level of urban morphology, especially from the macro perspective of urban morphology to study the urban summer thermal environment in severe cold regions. Therefore, in order to cope with the increased frequency of urban heat islands and high temperatures under a warming climate, it is necessary to propose adaptive spatial strategies for cold regions to cope with the summer thermal environment. Secondly, building forms have different mechanisms of influence on the thermal environment in various cities and climatic conditions, and it is necessary to understand the mechanisms of influence on the thermal environment and spatial form in further detail for each city. In addition, rivers are an important source of urban cooling and an important way to mitigate urban thermal environment, but the mechanisms of the river cooling effect in relation to urban morphology at an urban level are still under-researched and are often overlooked by urban planners when developing road, landscape and neighborhood morphology around rivers. It is therefore crucial to address the research related to the thermal environment and urban morphology around rivers in severe cold regions in order to identify strategies for the design of urban spaces.

Thermal environment studies in conjunction with LST have been applied in many cities (31, 32). This study takes the central area of Shenyang, a cold region of China, as an example. Typical high-density urban neighborhoods around the large river Hun River are selected for analysis, satellite inversion surface temperature and urban morphology data are calculated, linear regression models and spatial regression models are used to explore the cooling effect of the river and to analyses the relationship between urban morphology and surface temperature, and to provide guiding suggestions for adaptive design to cope with the high summer temperatures in Shenyang.

The main objectives of this study are as follows: (1) to analyses and verify the cooling effect and cooling range of the Hun River, and to provide a basis for urban strategies to exploit the cooling effect; (2) to calculate the correlation between urban morphological parameters and LST in the area around the Hun River and the spatial regression relationship, and to compare the mechanisms and differences in the thermal environment impact of different urban morphological parameters; (3) to propose corresponding response strategies. The results of the study provide a theoretical basis and technical guidance for spatial optimization of the use of rivers as cold sources in cold regions to mitigate the thermal environment in hot weather in the context of climate change.

## 2. Study area

**Figure 1:**
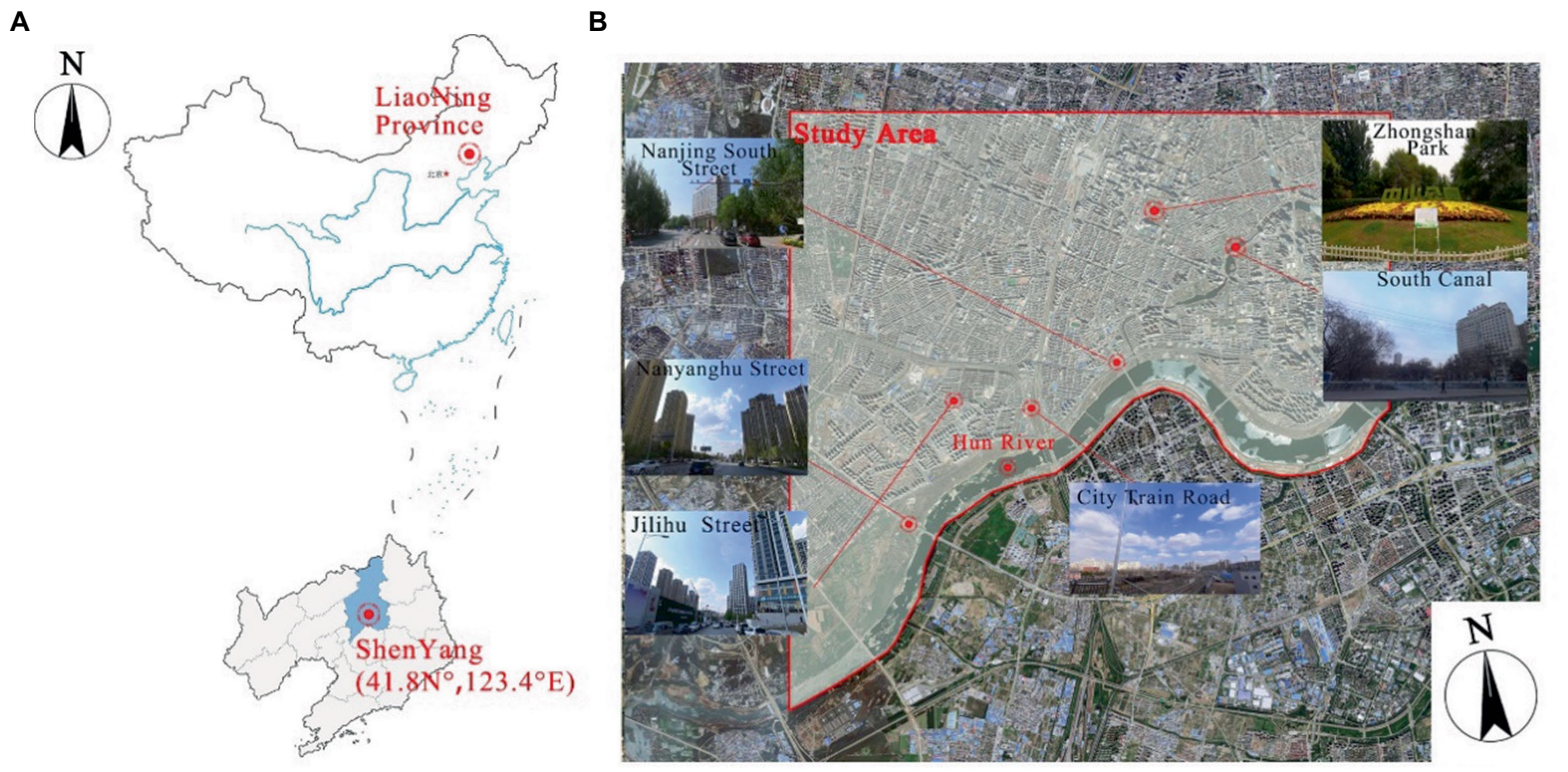
Study area.

The city of Shenyang, the subject of the study, is located in the northeast of China (N41°48′11.75″, E123°25′31.18″) and has a temperate monsoon climate with distinctive climatic features in all seasons, with the highest temperature occurring in July, when the average temperature is around 24°C and the hottest temperature can be as high as 39°C. The Hun River in Shenyang flows mainly from the northeast to the southwest and is an important landscape resource in the city. The study area is the surrounding cities in the main basin of the Hun River, with high-density urban neighborhoods and a rich variety of architectural forms (Figure 1). The study area includes large green areas such as the South Canal and Zhongshan Park, as well as a large number of residential and commercial areas, mainly the riverfront residential area, the station commercial area, the South Canal area and the financial and commercial area of Youth Street. There are four major urban roads perpendicular to the river in the study area, namely Nanyang Lake Street, Gili Lake I Street, City Train Road and Nanjing South Street.

## 3. Materials and methods

**Figure 2:**
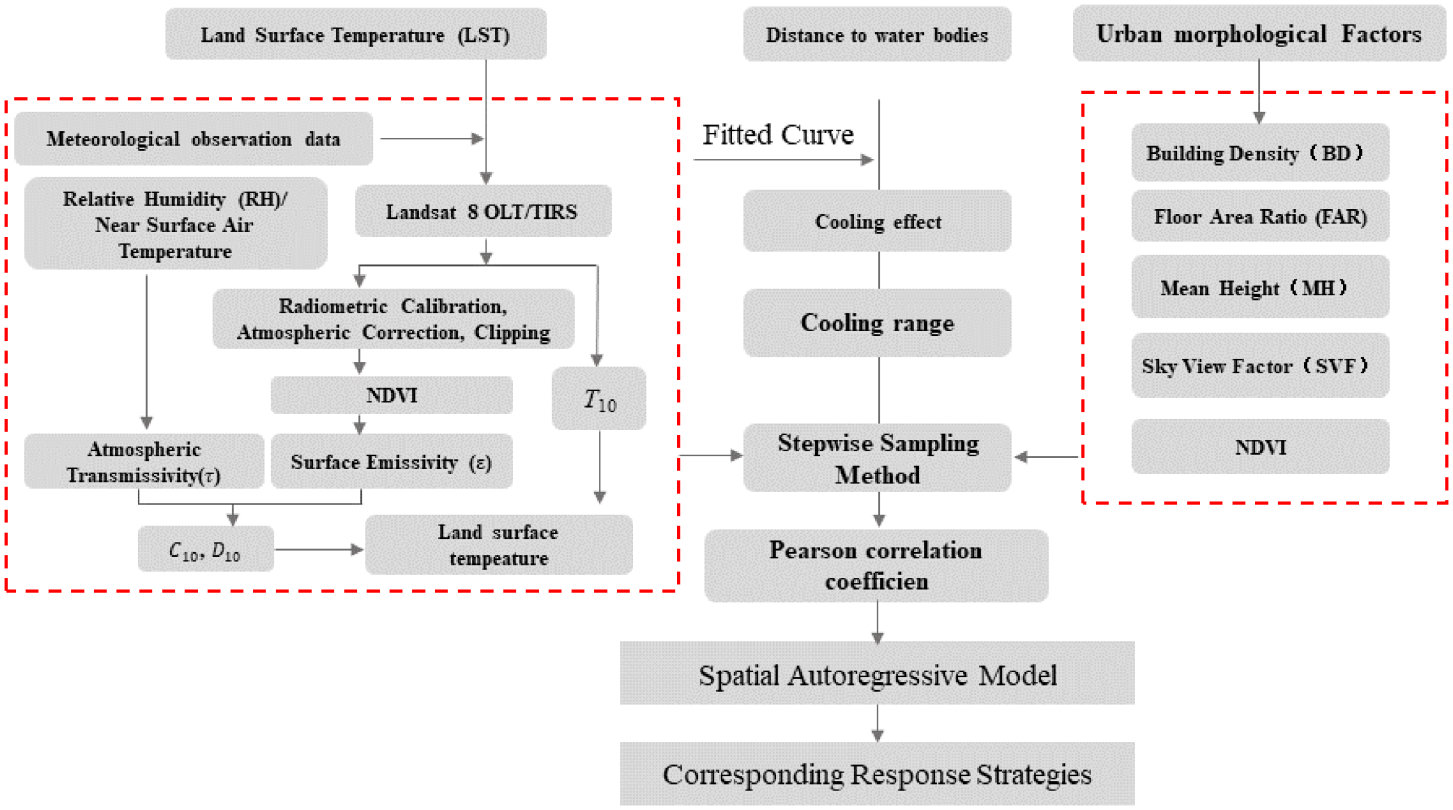
Research framework.

In this study, GIS, remote sensing data inversion, correlation analysis and spatial regression models were used to analyze and collate the basic data and calculate the urban heat island intensity, and finally, step-by-step sampling was used to verify the cooling effect of rivers and establish a spatial regression model, the research process and ideas are shown in Figure 2.

The remote sensing data in this study were downloaded from the Landsat8 satellite data from the Geospatial Data Cloud website^1^, and the remote sensing images were acquired on 26 August 2016 at 10:34 a.m. There was no cloud coverage over the study area, and the acquired remote sensing images were processed through the ENVI software platform for data calculation, and the surface temperature A single window algorithm was used for the inversion, which in turn was used to obtain data such as surface temperature and normalized vegetation index (33).

Spatial scale (grid size) is a decisive variable in urban environmental studies. The urban surface has a complex built form and if the scale is too large it will affect the accuracy of the data, while if the scale is too small it will not be representative of the whole area. Therefore, based on the study by Masson, Nassar and others, a scale of 250 × 250 m is used for the study of neighborhoods, which is a good representation of urban morphology and also correlates well with the local microclimate of the city (34, 35).

### 3.1. Inversion of land surface temperature

**Figure 3:**
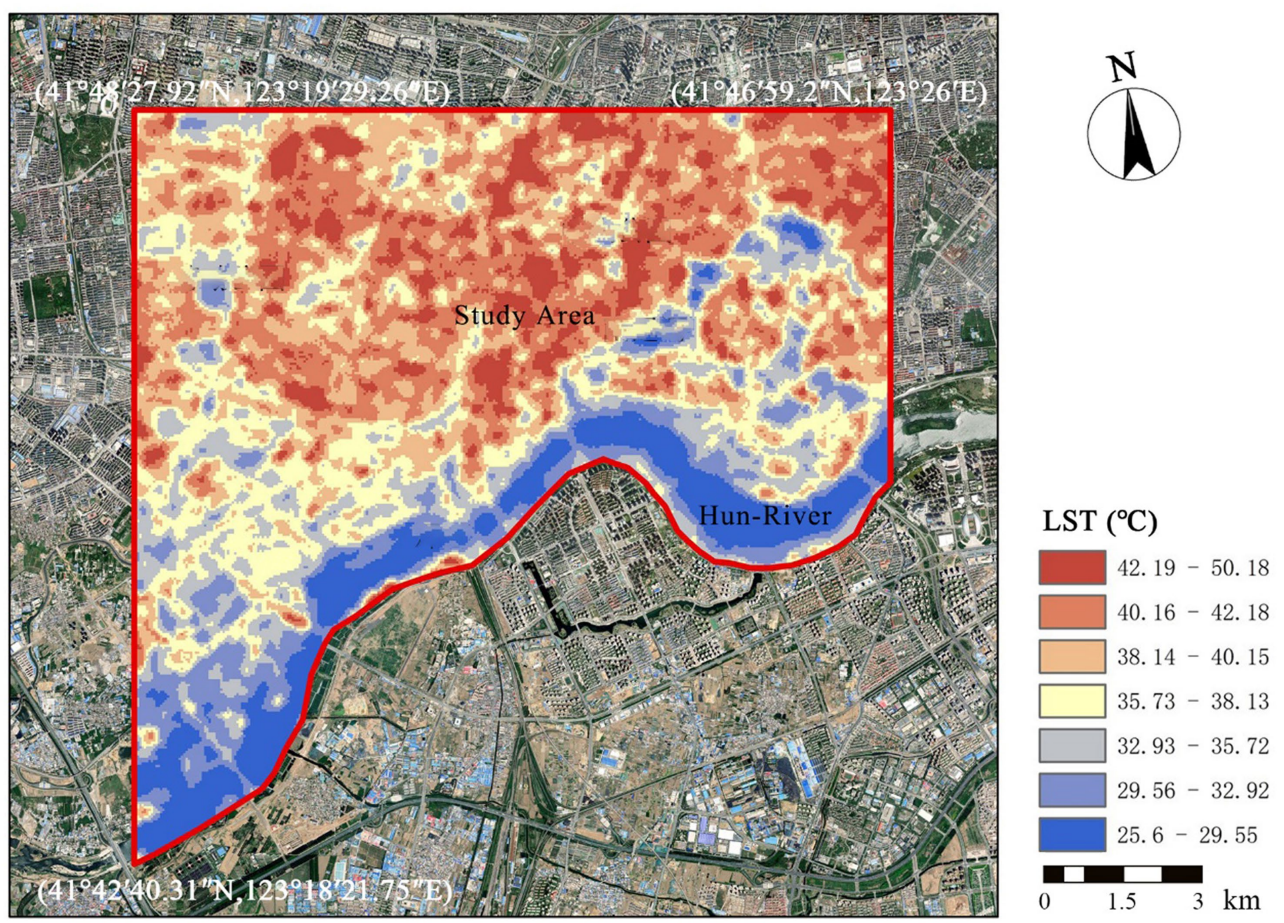
Land temperature distribution map.

Based on the study area boundaries and data sources, we mapped Landsat 8 thermal infrared sensor (IRS) images within the study area, representing the LST distribution during heat and non-heat waves, respectively (Figure 3).

Common methods used to calculate the LST are the single-window algorithm, the split-window algorithm and the single-channel algorithm. The single-window algorithm is a function of atmospheric transmittance (τ), mean atmospheric temperature (Ta) and surface emissivity (ε). The parameters used in this method are easily obtained and the spatial distribution of the LST can be accurately obtained. In summary, the single-window algorithm proposed by Qin Zhihao et al. is used in this paper to calculate the LST (Eq. 1) (33).


(1) $$T_{\mathrm{surface}}=\frac{a_{10}\left(1-C_{10}-D_{10}\right)+\left(\begin{array}{l} b_{10}\left(1-C_{10}-D_{10}\right)\\ +C_{10}+D_{10} \end{array}\right)T_{10}-D_{10}T_{a}}{C_{10}}\;$$

(2) $$C_{10}=\varepsilon \tau \;$$

(3) $$D_{10}=\left(1-\tau \right)\times \left(1+\left(1-\varepsilon \right)\times \tau \right)\;$$

Based on the temperature range of the study area, a coefficient value of −62.7182 and a b value of 0.4339 were selected for this study. T_10_ is the brightness temperature of Landsat 8 TIRS Band 10. C_10_ and D_10_ are the internal parameters of the algorithm based on atmospheric parameters and ground radiance. The relative humidity and near-surface air temperature entered in the single-window algorithm were obtained from meteorological observations during the study period. Finally, the image raster was converted to 100 m using the GIS resampling tool to match other data layers. The LST values obtained in this way were the dependent variables for the quantitative analysis.

### 3.2. Urban spatial factors

In this study, various urban morphology and cold source factors were selected to describe the spatial conditions of the city. The urban morphology factors include NDVI (Normalized difference vegetation index), FAR (Floor area ratio), SVF (Sky view factor), MH (Building mean height) and BD (Building density). The calculation formulae are shown in Table 1.

**Table 1:** Definition and calculation of urban spatial factors.

Factor	Formula/Calculation	
BD	$BD=\frac{\sum_{i=1}^{n}S_{i}}{S_{A}}\;$	$S_{i}$: basal area of a single building $S_{A}$: land area $h_{i}:$ building height $F_{i}$: number of floors on a single building
FAR	$FAR=\frac{\sum_{i=1}^{n}S_{i}F_{i}}{S_{A}}$	
MH	$MH=\frac{\sum_{i=1}^{n}S_{i}h_{i}}{\sum_{i=1}^{n}S_{i}}$	
SVF	$SVF=1-\overset{n}{\underset{i=1}{\sum }}\sin^{2}\beta \left(\frac{a_{i}}{360{}^{\circ}}\right)$	β: azimuth angle of i $a_{i}$: elevation angle of i
NDVI	$NDVI=\frac{NIR-R}{NIR+R}$	NIR is band 5 in Landsat8 data for the near infrared band and R is band 4 in Landsat8 data for the red light band.

### 3.3. River cooling effect

The cooling effect of water bodies was obtained by correlation analysis of urban morphological factors and surface temperature. A stepwise regression method was first used to gradually remove samples from the vicinity of the water body, and then correlation analysis was used to determine whether LST increased with distance and whether the correlation between urban morphological factors and LST varied with distance. Due to the large area of the Hun River and the large cooling range, 500 m was used as the distance unit and the sample was gradually excluded eight times, with the furthest distance being 4,000 m. A linear regression model of the urban morphological parameters as variables and the urban surface temperature was established by correlation analysis, while the standardized regression coefficient *R*^2^ was also used to explain and compare the degree of influence of the factors. The Pearson correlation coefficient was compared with the *R*^2^ value to analyses whether the cooling effect of the water body affects the relationship between the urban morphological factor and LST and how far it can reach at maximum.

### 3.4. Spatial regression models

According to Tobler’s first law “Everything is correlated with everything else, but things that are close together are more correlated than things that are distant.” It can be assumed that surface temperatures may have spatial autocorrelation. The spatial autocorrelation of surface temperature (LST) may be explained by the fact that surface temperatures interact with each other between spatial units due to heat conduction, convection and radiation. This study therefore builds spatial autoregressive models using GeoDa, a free, open-source software tool that can support the building of multiple spatial autoregressive models. Therefore, before building the spatial regression model, the global Moran’s I and the local Moran’s I were used to test the global spatial autocorrelation and the local spatial autocorrelation of the surface temperature (LST) to verify the spatial autocorrelation of the LST, respectively. A Moran’s I greater than 0 indicates a positive correlation and less than 0 indicates a negative correlation; a value close to 0 indicates that the variables are randomly distributed or that there is no spatial autocorrelation; a value close to 1 indicates a perfectly positive correlation; a value close to −1 indicates that dissimilar properties are clustered, i.e., high values are adjacent to low values.

Two types of spatial regression models are often used in spatial regression analysis: the Spatial Lag Model (SLM) and the Spatial Error Model (SEM). The Spatial Lag Model (SLM) is considered to be the result of autocorrelation of the dependent variable, which is considered in the dependent variable equation. In the field of research, the Spatial Lag Model (SLM) is more effective when the dependent variable within a cell is more likely to be influenced by the dependent variable in nearby cells, and the Spatial Error Model (SEM) is more effective when the error term of the variable is more likely to be influenced by the spatial location. The expressions for the spatial lag model SLM and the spatial error model SEM are as follows, respectively:


(4) $$y_{SLM}=\rho W_{y}+x\beta +\varepsilon \;$$

(5) $$y_{SEM}=x\beta +\gamma W_{\varepsilon }+\delta \;$$

where *ρ* is the spatial correlation coefficient, ***W***_*y*_ is the spatial matrix of the dependent variable, *β* the independent variable coefficient, *γ* is the residual correlation coefficient, ***W***_*ε*_ is the residual spatial matrix, and *ℇ* is the residual variable. The independent variables in this study are sky visibility factor (SVF), building density (BD), mean building height (MH), floor area ratio (FAR), normalized difference vegetation index (NDVI), and the dependent variable is LST.

The Lagrange Multiplier (LM) and Robust Lagrange Multiplier (R-LM) methods were used to test the variables to select the appropriate model for the study. The judgment is based on comparing the magnitude of LM (lags) and LM (errors), R- LM (errors) and R-LM (errors) while ensuring significance. Where LM (lag) and R- LM (lag) represent the spatial lag model (SLM) and LM (error) and R-LM (error) represent the spatial error model (SEM). The spatial lag model (SLM) is chosen when LM (lag) and R-LM (lag) are large (or significant), and the spatial error model (SEM) when LM (error) and R-LM (error) are large.

## 4. Results and discussion

### 4.1. Cooling effect influence mechanism

**Figure 4:**
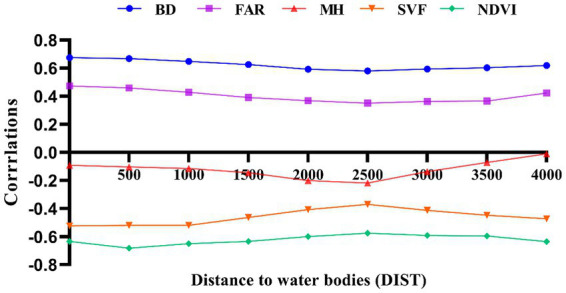
Pearson correlation coefficient LST— urban morphological factors.

The Pearson correlation coefficients between each morphological parameter and LST were calculated by SPSS software with the change of distance, and the calculated results were statistically analyzed as shown in Figure 4. When 0 < DIST<2,500 m, the correlation between urban morphological factors and LST remains low and stable. When DIST>2,500 m, the correlation between some urban morphological factors and LST increases rapidly, such as the significant negative correlation between NDVI, SVF and LST, but this correlation increases inversely after 2,500 m. As a major factor reflecting open space and shading effectiveness, a higher SVF usually means that there are fewer buildings or low-rise urban forms that can reach the surface, and therefore, a higher SVF leads to a higher LST during the day. However, by comparing the spatial distribution of the samples, i.e., LST and SVF values on each distance interval, the analysis revealed that the samples with high SVF values and low LST values were mostly distributed in areas near the river. This is because open areas are more susceptible to the influence of water bodies. Open space facilitates air flow and accelerates heat exchange, which has a significant cooling effect on the ground surface and weakens the positive correlation between SVF and LST. As the distance increases, the cool airflow from the water body is gradually heated by the surface or buildings, and the cooling effect diminishes, weakening the influence of the water body on more distant areas, and the positive correlation between SVF and LST increases.

In Figure 4, NDVI, a common indicator of vegetation cover, was significantly and negatively correlated with LST. The Pearson correlation coefficient decreased with increasing distance. The linear regression model between NDVI and LST became significant immediately after excluding samples at distances less than 2,500 m. We also found that surface temperatures corresponding to study units with the same NDVI values had lower LST values for samples close to water bodies, which also suggests that water bodies have a greater influence on the surrounding area, interfering with the correct quantitative relationship between NDVI and LST.

BD, FAR and LST are significantly positively correlated, and the correlation between BD, FAR and LST remains low and stable when DIST <2,500 m, and the correlation between the two parameters and LST increases inversely when DIST >2,500 m. The analysis found that regions with the same high FAR and BD would have lower corresponding LST along the river, because the cooling effect along the river would interfere with the positive correlation between FAR, BD and LST.

The correlation between MH and LST shows a negative correlation, as do SVF and FAR, but the Pearson correlation coefficient between MH and LST has remained low and not clearly correlated, but both change inversely at 2,500 m. It also indicates that the 2,500 m range is influenced by river cooling effects.

**Figure 5:**
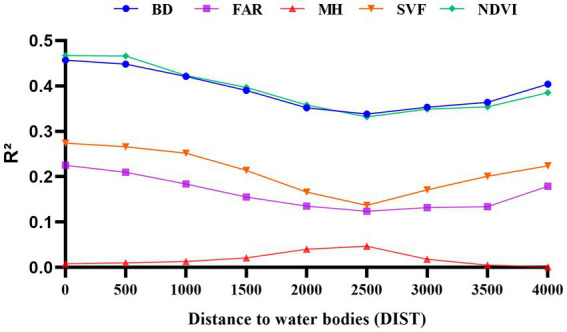
LST— R^2^ of fitting coefficients of urban morphological factors.

The fit coefficients R^2^ of the morphological parameters and LST were calculated using the stepwise sampling method, and a trend consistent with the Pearson correlation coefficients was found, with the inverse abrupt change occurring at 2,500 m. This reinforces the influence of the water-cooling effect on the relationship between the morphological parameters and LST within 2,500 m. The analysis found that BD and NDVI maintained a good fit with LST, with the coefficients R^2^ reaching 0.467 and 0.457 respectively, while FAR, SVF and MH had a poor fit with LST, with R^2^ remaining low Figure 5. It is worth noting that the correlation between individual parameters and LST appears to be below 0.2, with a minimum of less than 0.1. The same situation has been observed in similar previous studies (19). The main reason for this is that LST is influenced by a combination of factors, whereas the Pearson correlation coefficient only addresses the relationship between a single parameter and LST. This further suggests that LST is difficult to predict from a single parameter and that the effect of a combination of factors on LST needs to be further discussed with the help of a multiple regression model.

**Figure 6:**
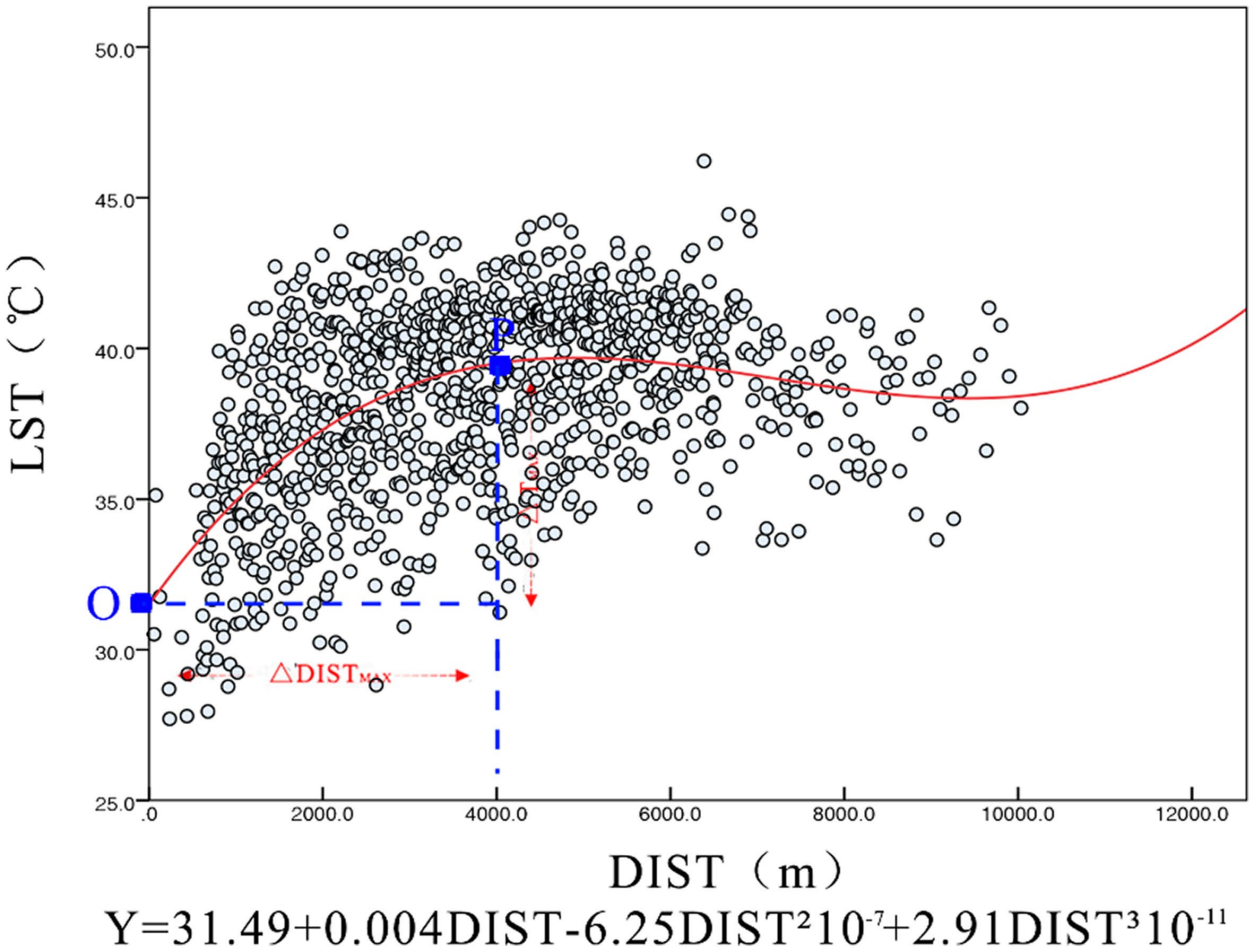
Scattered plot of offshore distance and temperature.

We performed a regression analysis Figure 6 on all data samples calculated and obtained a significant correlation distance of 4,000 m. Combining the Pearson correlation coefficient and the fitted coefficient R^2^ with the curvature change of the curve, we obtained an optimum cooling distance of 2,500 m with an associated maximum cooling temperature of 7°C. This also indicates that most of the study area is affected by the cooling effect of the Hun River.

### 4.2. Regression model analysis results

Spatial autocorrelation validation of LST in GeoDa, the Moran index is often used to validate the spatial autocorrelation of cells and is used to express the clustered distribution of variable data in the study cell (36). It is calculated as follows.


(6) $$I=\frac{n\sum_{i}\sum_{j}w_{ij}\left(x_{i}-\bar{x}\right)\left(x_{j}-\bar{x}\right)}{\left(\sum_{i}\sum_{j}w_{ij}\right)\sum_{i}{\left(x_{i}-\bar{x}\right)}^{2}}\;$$

where n is the geographical unit; x*_i_* and x*_j_* are the spatial locations of i and j; and w*_ij_* is the spatial weight of the spatial structure between units *i* and *j*. The Moran’s I result of 0.683 was calculated by the software and was significantly correlated, indicating the presence of LST with strong spatial autocorrelation. Experiments can therefore be conducted using the spatial autoregressive model (SAR) technique.

In order to select the appropriate type of spatial autoregressive model (SAR) prior to the experiment, the values of LM(lag) and LM(error), R- LM(lag) and R-LM(error) were calculated using the analysis software GeoDa, and then the magnitudes of the two sets of data were compared to determine the choice of model. Testing nine models for the stepwise sampling method using GeoDa (Table 2). The results of the LM analysis showed that nine models had a *p*-value of 0 and eight models had a value of LM(lag) greater than LM(error), then comparing the test values of R-LM, again all had a *p*-value of 0 and all eight models had a value of R-LM(lag) greater than R-LM(error), with only one model having the opposite. Based on the test results, the SLM and SEM models were selected for the spatial regression analysis of the corresponding data. The SLM model was used for the eight models from 0 to 3,500 m, and the SEM model was used for the model in the 4,000 m range.

**Table 2:** Lagrange multiplier method for spatial dependence diagnosis (model 1–model 9).

	0	500	1,000	1,500	2,000
Lagrange multiplier (lag)	**702.4156**	**675.4082**	**667.5104**	**588.1220**	**517.0403**
Robust LM (lag)	**145.2238**	**130.9775**	**135.4465**	**123.1254**	**110.8589**
Lagrange multiplier (error)	638.2214	641.3269	613.6186	528.9586	451.9600
Robust LM (error)	81.0296	96.8961	81.5747	63.9620	45.7786
	**2,500**	**3,000**	**3,500**	**4,000**	
Lagrange multiplier (lag)	**451.5896**	**360.1648**	**310.5523**	232.1848	
Robust LM (lag)	**94.8063**	**68.9224**	**60.7878**	29.6346	
Lagrange multiplier (error)	399.5371	328.4569	277.0409	**241.0755**	
Robust LM (error)	42.7537	37.2144	27.2764	**38.5253**	

In the results of tables, the values marked with * represent values with some correlation and the values bolded and marked with * represent values with significant correlation.

The four parameters, R^2^, the fit coefficient, AIC (Akaike Information Criterion), LL (Log Likelihood) and BIC (Bayesian Information Criterion), calculated by GeoDa, can describe the goodness of fit of each model, and can determine the explanatory power of a parameter or a combination of parameters for LST.

The larger the values of R^2^ and LL and the smaller the values of AIC and BIC, the better the fit of the model. Both models maintain a high level of R^2^ after simulation, indicating that the change in temperature is a combination of multiple factors. As the distance changes from 0 to 4,000 m, the values of R^2^ and LL are gradually larger and the values of AIC and BIC are gradually smaller, this change indicates that the model fit is getting better and better, indicating that the distance has a great influence on the relationship between each morphological parameter and LST, and also reflects the river cooling effect on temperature.

The results of the calculation in Table 3 show that the nine regression coefficients R^2^ have been maintained at a high level, up to 0.77. Compared with the traditional regression model, the spatial regression model avoids over-expanding the influence of one morphological parameter on the surface temperature, and can better analyses the relationship between each morphological parameter and the surface temperature; With the change of distance, the morphological parameters of each city fluctuate relatively more in the range of 0 ~ 2,500 m, and stabilizes after 2,500 m, which is consistent with the results of previous analysis using traditional models. This result further verifies that the Hun River has a strong influence on the correlation between various urban morphological factors and LST in the range of 2,500 m.

**Table 3:** SLM and SEM model results summary.

Explanatory variables	0	500	1,000	1,500	2,000
FAR	−0.621923^*^	−0.592474^*^	−0.558484^*^	−0.581561^*^	−0.639688^*^
NDVI	−9.14571^*^	−11.9261^*^	−11.0686^*^	−10.6083^*^	−11.5172^*^
SVF	−1.30675^*^	−0.903103^*^	−0.794795^*^	−0.743754^*^	−0.905655^*^
MH	−0.015952^*^	−0.020362^*^	−0.023883^*^	−0.0225889	−0.020622^*^
BD	6.48366^*^	5.46208^*^	5.70278^*^	5.96807^*^	5.96936^*^
R^2^	0.774048^*^	0.773820^*^	0.771452^*^	0.756536^*^	0.732695^*^
LL	−1967.71	−1911.94	−1839.18	−1688.55	−1538.27
AIC	3949.43	3837.89	3692.35	3391.1	3090.54
SC	3984.36	3872.74	3726.97	3425.16	3123.92
	**2,500**	**3,000**	**3,500**	**4,000**	
FAR	−0.564231^*^	−0.567791^*^	−0.757822^*^	−0.792847^*^	
NDVI	−11.7415^*^	−12.2787^*^	−10.5721^*^	−14.8075^*^	
SVF	−1.27097^*^	**−1.31858**	**−0.986023**	**−1.59811**	
MH	0.0361844^*^	**−0.0386267**	**−0.0214078**	**−0.0295309**	
BD	5.27814^*^	5.54606^*^	7.5223^*^	8.55264^*^	
R^2^	0.732120^*^	0.718081^*^	0.716654^*^	0.724359^*^	
LL	−1355.45	−1171.87	−1011.03	−848.99712	
AIC	2724.9	2357.74	2036.05	1709.99	
SC	2757.52	2389.44	2066.74	1735.27	

In the results of tables, the values marked with * represent values with some correlation and the values bolded and marked with * represent values with significant correlation.The * sign indicates that the numerical results are somewhat correlated, but not significantly so.

The results of the analysis of the nine models show that FAR, NDVI and SVF are negatively correlated with LST, while BD is positively correlated with LST. NDVI accounts for the largest proportion of the models, while FAR accounts for the smallest. This also shows that NDVI has a greater influence on LST, greenery can absorb thermal radiation well and also improve the thermal environment of the study area, which has a greater potential to reduce the temperature of the study area; SVF tends to be related to the solar radiation received, and areas with a large SVF will receive more solar radiation and in turn will have higher surface temperatures, but the SVF in this study is a relatively small proportion of the overall regression model and has a relatively weak effect on the surface temperature of the whole area.

BD accounts for a relatively large proportion of each regression model parameter and has a large impact on LST; study areas with high density buildings tend to have poor ventilation, less green permeable surfaces, and large impervious surfaces are able to absorb a large amount of radiation during the day, causing a sharp increase in temperature in the study area and seriously affecting living comfort; this has similarities to the conclusions of other scholars under other climatic regions in China that urban density and height are the main factors affecting the urban thermal environment (37–39). High temperatures in urban neighborhoods caused by high building density can be mitigated by adding vegetation to the building envelope and changing the substrate (40, 41). In addition, BH, although showing a negative correlation, is a very small proportion of the model and the correlation is very weak, indicating that the influence of MH on surface temperature LST is not significant. The above findings reveal the main urban morphological factors affecting the urban thermal environment in the northern cold regions.

### 4.3. Limitations

This study objectively analyses the influence mechanisms of rivers on the surrounding environment mainly from the perspective of urban morphological factors, but there are still some limitations in the study. For example, there are many factors affecting the urban thermal environment, such as the cooling effect of water bodies on the urban thermal environment, which is related to sunlight, ventilation, etc. The influence of the evaporation of river water on the surrounding environment under different sunlight conditions; the different cooling effects of different wind directions on different neighborhoods. In the future, we will consider more influencing factors to improve the accuracy of the study, such as setting up multiple measurement points to investigate the causes of temperature differences in different regions, and adding climate and wind direction to further explore the influencing factors.

## 5. Conclusion

This paper presents a spatial regression model analysis of the environment around the Hun River in Shenyang. The spatial regression model calculation between LST and urban morphological factors verifies the mechanism of the cooling effect of the river on the surrounding neighborhoods and finds the following conclusions.

1. The river has a cooling effect on the surrounding urban neighborhoods and distance has a direct influence on the relationship between various urban form factors and LST, with a certain regularity in terms of distance.
2. Using the step-by-step sampling method of analysis, the maximum cooling distance of the Hun River for the surrounding environment is 4,000 m, but the optimum cooling intensity distance is 2,500 m.
3. Based on the discussion of the results of the analysis of different slices by stepwise sampling, we find that there is an interaction between some of the parameters LST in the urban morphological factors with the change of distance. For example, the influence of NDVI and BD on LST starts to gradually increase after 2,500 m, indicating that the influence of the river on the surrounding cooling effect gradually decreases after 2,500 m.

This paper analyses urban surface temperature change from both an urban morphology and river perspective, in order to investigate the extent and pattern of river influence on surrounding urban neighborhoods. The above research findings provide a case reference for the mechanism of the influence of urban water bodies on the surrounding environment in cold regions, and provide data support for urban management, planning and architectural design work. In future urban planning and design, the cooling effect mechanism of rivers can be fully utilized in planning and design, and measures such as controlling building form, increasing green vegetation cover and reducing building density can be taken to improve the urban thermal environment and reduce urban heat island.

## Data availability statement

The original contributions presented in the study are included in the article/supplementary material, further inquiries can be directed to the corresponding authors.

## Author contributions

FG, SX, and JZ conducted the experiments, analyzed the data, and wrote the manuscript. HZ and LL conceived and designed the study, supervised the research, and revised the manuscript. ZZ and XY assisted with the experiments and data analysis. All authors reviewed and approved the final manuscript.

## Funding

This research was supported by the Fundamental Research Funds for the Central Universities No. DUT21RW204 and the National Natural Science Foundation of China (Grant Nos. 52108044 and 52208045).

## Conflict of interest

The authors declare that the research was conducted in the absence of any commercial or financial relationships that could be construed as a potential conflict of interest.

## Publisher’s note

All claims expressed in this article are solely those of the authors and do not necessarily represent those of their affiliated organizations, or those of the publisher, the editors and the reviewers. Any product that may be evaluated in this article, or claim that may be made by its manufacturer, is not guaranteed or endorsed by the publisher.

## Figures and Tables

**Table:** 

Name	Definition
LST (Land surface temperature)	Indicator used to characterize the surface temperature of a city.
NDVI (Normalized difference vegetation index)	A normalization index that describes the spatial distribution of vegetation, and the range is −1–1.
FAR (Floor area ratio)	An urban form factor that represents the ratio of overall floorage to research unit.
SVF (Sky view factor)	The ratio at a point in space between the visible sky and a hemisphere centered over the analyzed location.
MH (Building mean height)	An urban form factor that represents the ratio of total building volume to total building base area.
BD (Building density)	The floor area of the building divided by the total area of the site.

## Footnotes

^1^ http://www.gscloud.cn
